# Dosimetry of ^177^Lu-PSMA-617 after Mannitol Infusion and Glutamate Tablet Administration: Preliminary Results of EUDRACT/RSO 2016-002732-32 IRST Protocol

**DOI:** 10.3390/molecules24030621

**Published:** 2019-02-11

**Authors:** Anna Sarnelli, Maria Luisa Belli, Valentina Di Iorio, Emilio Mezzenga, Monica Celli, Stefano Severi, Elisa Tardelli, Silvia Nicolini, Devil Oboldi, Licia Uccelli, Corrado Cittanti, Manuela Monti, Mahila Ferrari, Giovanni Paganelli

**Affiliations:** 1 Medical Physics Unit, Istituto Scientifico Romagnolo per lo Studio e la Cura dei Tumori (IRST) IRCCS, 47014 Meldola, Italy; anna.sarnelli@irst.emr.it (A.S.); emilio.mezzenga@irst.emr.it (E.M.); 2 Oncology Pharmacy, Istituto Scientifico Romagnolo per lo Studio e la Cura dei Tumori (IRST) IRCCS, 47014 Meldola, Italy; valentina.diiorio@irst.emr.it; 3 Nuclear Medicine Unit, Istituto Scientifico Romagnolo per lo Studio e la Cura dei Tumori (IRST) IRCCS, 47014 Meldola, Italy; monica.celli@irst.emr.it (M.C.); stefano.severi@irst.emr.it (S.S.); elisa.tardelli@irst.emr.it (E.T.); silvia.nicolini@irst.emr.it (S.N.); giovanni.paganelli@irst.emr.it (G.P.); 4 Radiology Unit, Istituto Scientifico Romagnolo per lo Studio e la Cura dei Tumori (IRST) IRCCS, 47014 Meldola, Italy; devil.oboldi@irst.emr.it; 5 Diagnostic Imaging Unit—Morphology, Surgery and Experimental Medicine Department, University of Ferrara, 44121 Ferrara, Italy; licia.uccelli@unife.it (L.U.); corrado.cittanti@unife.it (C.C.); 6 Unit of Biostatistics and Clinical Trials, Istituto Scientifico Romagnolo per lo Studio e la Cura dei Tumori (IRST) IRCCS, 47014 Meldola, Italy; manuela.monti@irst.emr.it; 7 Physics, IEO, European Institute of Oncology IRCCS, 20141 Milan, Italy; mahila.ferrari@ieo.it

**Keywords:** theragnostic tracers, PSMA, protectors, dosimetry

## Abstract

Radio-ligand therapy (RLT) with^177^Lu-PSMA-617 is a promising option for patients with metastatic castration-resistant prostate-cancer (mCRPC). A prospective phase-II study (EUDRACT/RSO,2016-002732-32) on mCRPC is ongoing at IRST (Meldola, Italy). A total of 9 patients (median age: 68 y, range: 53–85) were enrolled for dosimetry evaluation of parotid glands (PGs), kidneys, red marrow (RM) and whole body (WB). Folic polyglutamate tablets were orally administered as PGs protectors and 500 mL of a 10% mannitol solution was intravenously infused to reduce kidney uptake. The whole body planar image (WBI) and blood samples were acquired at different times post infusion (1 h, 16–24 h, 36–48 h and 120 h). Dose calculation was performed with MIRD formalism (OLINDA/EXM software). The median effective half-life was 33.0 h (range: 25.6–60.7) for PGs, 31.4 h (12.2–80.6) for kidneys, 8.2 h (2.5–14.7) for RM and 40.1 h (31.6–79.7) for WB. The median doses were 0.48 mGy/MBq (range: 0.33–2.63) for PGs, 0.70 mGy/MBq (0.26–1.07) for kidneys, 0.044 mGy/MBq (0.023–0.067) for RM and 0.04 mGy/MBq (0.02–0.11) for WB. A comparison with previously published dosimetric data was performed and a significant difference was found for PGs while no significant difference was observed for the kidneys. For PGs, the possibility of reducing uptake by administering glutamate tablets during RLT seems feasible while further research is warranted for a more focused evaluation of the reduction in kidney uptake.

## 1. Introduction

The most frequent cancer in adult males is prostate cancer (PCa). Prognosis is dependent on the tumor stage and is poor in patients with metastatic disease (mPC) as it has a five-year survival of only 29% [1]. Limited treatment options are available for the subgroup of metastatic patients with castration-resistant disease (mCRPC). The currently available treatment options are taxane-based chemotherapies (e.g., docetaxel, cabazitaxel) and novel second-line hormone therapies (e.g., enzalutamide, abiterone), which are all associated with moderate survival and poor quality of life [2,3].

Radioligand therapy (RLT), which is based on a combination of a short-range energy radionuclide and a substrate with high specificity for cancer cell receptors, enables lesions to be treated with targeted radiation. ^177^Lu is a short energy beta emitter with a maximum range in water of 1.9 mm and a half-life of 6.71 days. Prostate-specific membrane antigen (PSMA) is a protein overexpressed in 90–100% of local PCa lesions and metastatic disease (lymph node and bone). There is an even greater level of overexpression in high-grade mCRPC tumors [4]. In recent years, different RLT radiopharmaceuticals exploiting PSMA-targeting radioligands have been developed, among which the novel theragnostic ^177^Lu-PSMA-617 [5] is considered to be one of the most promising, with high specificity for the tumor and moderate uptake in the whole body and organs at risk (OaR). Mild toxicity has mainly been observed in patients undergoing ^177^Lu-PSMA, with around 10% experiencing adverse events [6]. However, the absorbed dose to the OaRs (kidneys, parotid glands and red marrow) [7,8,9] limits the maximum injectable activity, reducing the dose to the tumor and compromising therapeutic efficacy [6]. OaR drug protectors with high specificity for PSMA-ligand are thus needed to reduce off-target uptake in both parotid glands and kidneys.

With regard to parotid glands, an external ice pack cooling strategy was used by van Kalmthout et al. with the aim of reducing hematic flow and therefore local uptake [10]. A reduction in ^68^Ga-HBED-CC-PSMA-11 uptake in externally cooled salivary glands compared to non-cooled ones was observed in terms of maximum standard uptake values (SUV_max_) in PET images (14.52% reduction, 11.07 ± 3.53 versus 12.95 ± 4.16; *p*-value = 0.02) [10]. Although the external cooling technique seems to be a promising tool to reduce PSMA uptake in PET imaging, there is still no evidence of a reduced dose in parotid glands after treatment with ^177^Lu-PSMA-617.

Similarly, in peptide radionuclide receptor therapy (PRRT) for neuroendocrine tumors, a mean reduction of 27% (range 9–53%) in kidney uptake was observed with the infusion of an amino acid solution [11,12]. The reduction further increased to 39% when the infusion was prolonged for 10 h and finally reached 65% when prolonged for 2 days after injection [13]. The same strategy was used for ^177^Lu-DOTA-PSMA treatment [14]. Nevertheless, given the specific interaction of each ligand used as a vector for ^177^Lu molecule, the kinetic uptake and process of fixation may vary among treatment methods [14]. Unlike PPRT with somatostatin analogs, an efficient pharmacological method for nephroprotection has still not yet been found for RLT with PSMA inhibitors [15].

In April 2017, a prospective protocol (EUDRACT/RSO 2016-002732-32) with ^177^Lu-PSMA-617 therapy was activated at IRST (Meldola, Italy) for patients with mCRPC. The protocol includes a dosimetry objective to perform pharmacokinetic and absorbed dose evaluations to determine their biodistribution to OaRs. The treatment is delivered in association with drug protectors for parotid glands and kidneys with specificity for PSMA receptor.

To preserve salivary glands, polyglutamate folates of plant origin are orally administrated to patients during treatment in the form of tablets. This protector is a substrate of PSMA and exploits the enzymatic activity of PSMA receptors and the release of glutamates. Consequently, the glutamates compete with the ^177^Lu-PSMA-617 for the active sites of PSMA in competition. Moreover, ice packs are also positioned on the parotid glands. 

The protocol includes the infusion of a 10% mannitol solution as a kidney protector. This is because PSMA is mainly expressed in proximal tubules [16]. Acting mainly on this region as an osmotic diuretic drug, mannitol is a potential candidate for kidney protection [17].

The efficacy of mannitol has previously been demonstrated with ^68^Ga-HBED-CC-PSMA-11 for imaging purposes, with a reduction in kidney uptake expressed in terms of SUV_max_ (range: 7.4–24.3%) [18]. According to the promising results obtained for PET imaging, dosimetry studies in patients treated with ^177^Lu-PSMA-617 are needed to confirm its role as a nephroprotector.

The present work summarizes the preliminary results of the dosimetric evaluation of parotid glands and kidneys according to the EUDRACT/RSO, 2016-002732-32 protocol. For the sake of completeness, the preliminary dosimetric data for the whole body, liver and red marrow are also reported.

## 2. Results

### 2.1. Patients and Treatment Characteristics

A total of 32 patients were enrolled in the protocol from April 2017 to March 2018. Dosimetry evaluation was performed on 9 patients (6 during the first cycle and 3 during the second). The main patient and treatment characteristics are summarized in Table 1. A variability of 10% in injected activity is accepted due to different measurement uncertainties. Regarding patient 8, based on clinical considerations (only 5 months from 75-year achievement, excellent performance status, low tumor load and high weight of 94 kg), an exception for administered activity was performed in order to increase tumor load uptake. This exception was communicated to the local Ethical Committee, underlining that the risk benefit ratio was positive. All patients received renal and parotid gland protectors as protocol indications.

### 2.2. Dosimetry Results

**Figure 1:**
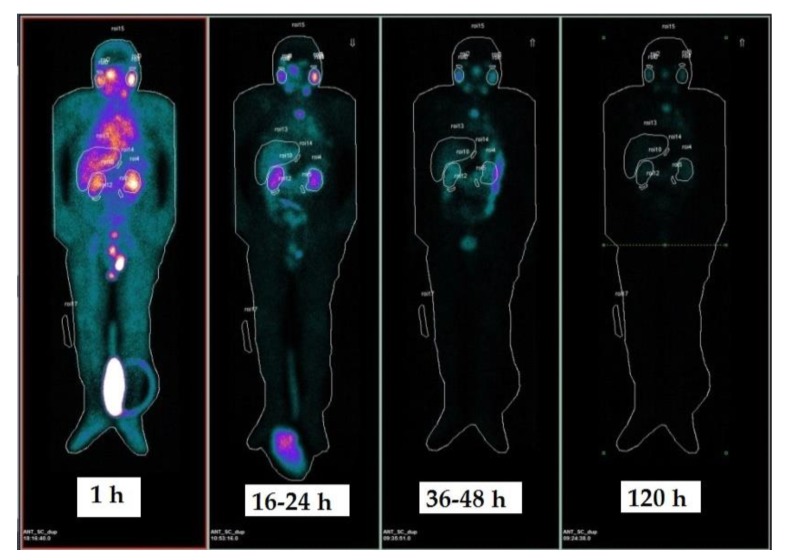
Sequential planar whole body images (WBI, anterior projection) acquired at 1 h, 16–24 h, 36–48 h and 120 h post infusion. Delineated organs: kidneys, parotid glands, liver, whole body. The first image (1 h) was acquired before bladder voiding and assumed as normalization point for injected activity.

Figure 1 shows an example of ROI contouring on anterior whole body images (WBI). Three acquisitions were only performed for patient no. 7, while red marrow dosimetry was performed in 6 patients. For parotid glands, a wash-in and wash-out trend was observed for all patients and a bi-exponential curve fitting was used. A maximum uptake was observed around 16 h after infusion for all patients. A combined wash-in/wash-out phase (4 patients) and pure wash-outs (5 patients) were observed for kidneys. Bi- and mono-exponential fitting models were used. In the cases of combined wash-in and wash-out phases, a maximum uptake was observed 16–24 h post infusion. With regard to the whole body and blood sample data for red marrow dosimetry, a pure wash-out trend was observed, which was fitted with a bi-exponential curve. Blood activity had already decreased by one order of magnitude compared to the initial blood activity 16 h post infusion.

The effective half-lives for all source organs are summarized in Table 2. The median values were 33.0 h (range 25.6–60.7) for parotid glands, 31.4 h (12.2–80.6) for kidneys, 25.4 h (12.5–62.9) for liver, 8.2 h (2.5–14.7) for red marrow and 40.1 h (31.6–79.7) for the whole body.

A transient high uptake in intestinal loops was observed at different times between 16 h and 120 h after infusion, with an important overlap over the kidneys. During the contouring phase, the overlap with high uptake intestine region over the kidneys was carefully avoided for each image. The counts of the partially-contoured kidney were then re-scaled to the whole kidney, assuming a uniform uptake between the overlapped and non-overlapped regions.

**Figure 2:**
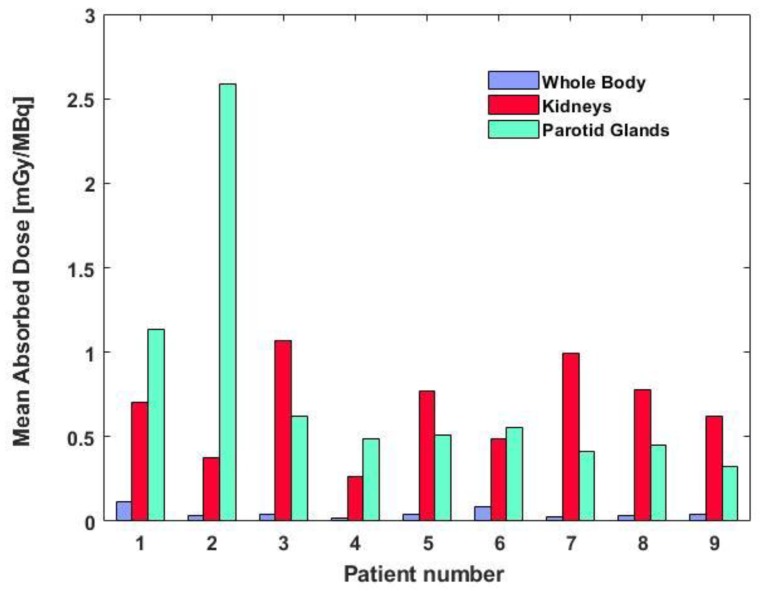
Whole body, kidney and parotid gland mean absorbed doses [mGy/MBq]. Whole body and kidney model was used in OLINDA/EXM software, while sphere model of unit density was used for parotid gland modeling.

The dosimetric results for our patient cohort are reported in Table 3 and Figure 2. The median values were 0.48 mGy/MBq (range 0.33–2.63) for parotid glands, 0.70 mGy/MBq (0.26–1.07) for kidneys, 0.13 mGy/MBq (0.05–0.53) for liver, 0.044 mGy/MBq (0.023–0.067) for red marrow and 0.04 mGy/MBq (0.02–0.11) for the whole body. Overall, homogeneity was observed among patients with the exception of parotid glands. The outlier value observed for patient no. 2 caused a higher standard deviation and larger range.

### 2.3. Comparison with Previous Studies

**Figure 3:**
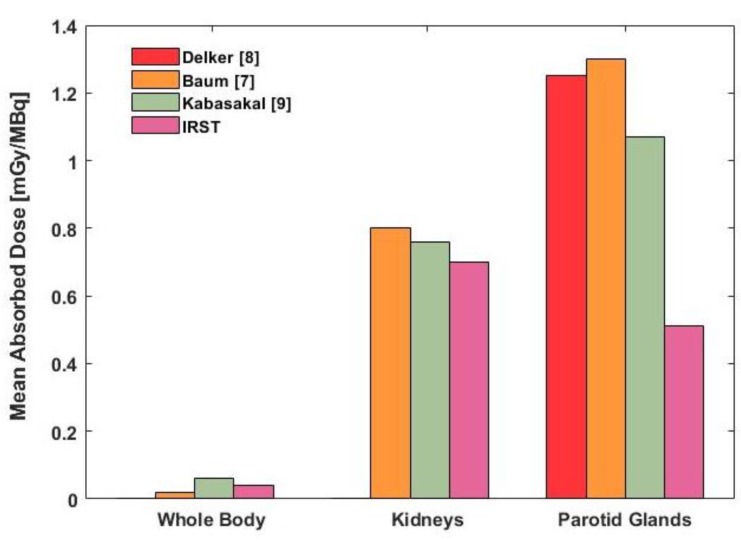
Comparison between our study data and previously published data in terms of median value.

**Figure 4:**
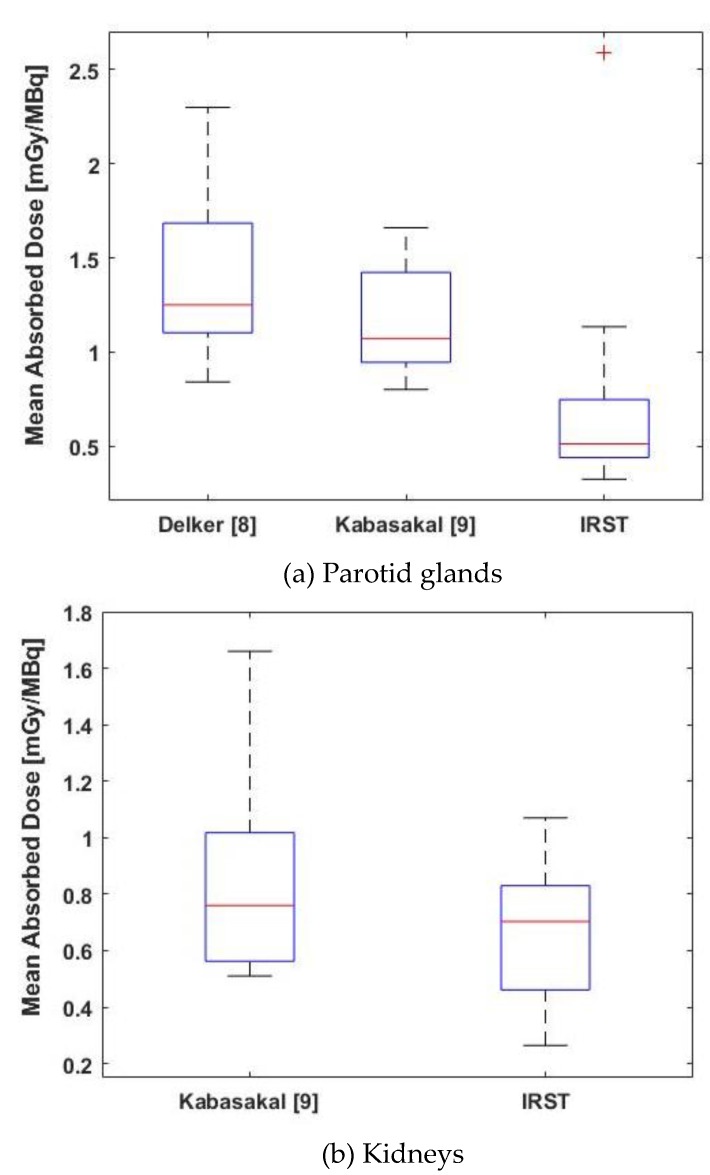
Box-plot comparison of dosimetric results between our study and previously published studies. (**a**) Parotid glands; and (**b**) Kidneys.

Detailed dosimetric data were available for kidneys and parotid glands from the studies by Delker [8] and Kabasakal [9], while median dose values were reported in Baum’s study [8]. Therefore, a graphical comparison (based on median and standard deviation data) was used for an overall comparison of the results (Figure 3), while a statistical comparison was performed for kidneys and parotid glands (Figure 4).

For parotid glands, a significant difference was observed between our data (median 0.48 mGy/MBq, range 0.33–2.63) and those of Kabasakal [9] (median = 1.07 mGy/MBq, range = 0.80–1.66) and Delker [8] (median = 1.25, range = 0.84–2.30). In the first comparison, the p-values for Mann-Whitney-Wilcoxon and Kolmogorov-Smirnov testes were 0.034 and 0.017, respectively. In the second comparison, the p-values were 0.045 and 0.041, respectively (Figure 4). Although a statistical comparison was not possible, a difference was also graphically visible with respect to Baum’s data [7], which had a median value of 1.3 mGy/MBq (range 0.3–9.5) (Figure 3).

A slight reduction in kidney doses in all patients was observed compared to literature data [8] (median = 0.8 mGy/MBq, range = 0.2–1.9). However, no significant difference was observed between our data (median = 0.70 mGy/MBq, range = 0.26–1.07) and those of Kabasakal [9] (median = 0.76, range = 0.51–1.66), with a *p*-value = 0.470 for Mann-Whitney-Wilcoxon test and a *p*-value = 0.648 for Kolmogorov-Smirnov test) (Figure 4).

## 3. Discussion

RLT with ^177^Lu-PSMA-617 has shown encouraging results for the treatment of mCRPC as it has high uptake in disseminated lesions [6,20]. A reduction in disease burden is obtained after repeated treatment cycles (sometimes even after the first one) in the majority of patients [5,21]. However, high PSMA uptake in specific OaRs, such as salivary glands and kidneys, may impair treatment efficacy by limiting maximum injectable activity [4]. Although severe salivary gland toxicities are now seldom reported for ^177^Lu-PSMA-617 treatment, such effects may occur very frequently when more advanced treatment techniques based on an α-particle (e.g., ^225^Ac-PSMA-617) are used. Kratochwil et al. described severe xerostomia patients treated as a last curative treatment option with ^225^Ac-PSMA-617 [22]. In more advanced treatment protocols where α-emitter PSMA-based drugs are used, parotid glands are the main OaRs and the administration of highly-specific organ protectors is an essential safety precaution [23,24]. With regard to renal toxicity, limited follow-up information is available for patients undergoing a last-chance treatment option due to their severely compromised baseline status. This may mask the onset of renal toxicity starting around 2 years after treatment [25]. Thus, when considering whether to start RLT earlier in an attempt to increase tumor control and overall survival, the potential for salivary gland and renal toxicity should be considered and attention should be paid to the OaR absorbed dose.

In our protocol, we focused on organ protectors with specificity for PSMA receptors and aimed to reduce the ^177^Lu-PSMA-617 uptake in these organs. The drug protection used for salivary glands (folic polyglutamate tablets; candies) is a substrate for PSMA receptors and the underlying strategy was to keep the PSMA enzyme active sites busy and thus, reduce the available binding sites for ^177^Lu-PSMA-617 fixation after intravenous infusion.

Our results revealed a significant reduction in parotid gland uptake in terms of mean absorbed dose compared to the literature data (median = 0.48 mGy/MBq [range = 0.33–2.63]). The Mann-Whitney-Wilcoxon *p*-values were 0.034 and 0.045 with Kabasakal [9] median of 1.07 mGy/MBq [0.80–1.66] and Delker [8] median of 1.25 [0.84–2.30], respectively.

At the renal level, mannitol acts as an osmotic agent in the proximal tubule and thus, the fixation of ^177^Lu-PSMA-617 at the proximal tubules may be decreased, reducing the kidney uptake. However, in this preliminary study, no significant difference was observed in terms of kidney absorbed dose, with a median value of 0.70 mGy/MBq (range = 0.26–1.07).

At Johns Hopkins School of Medicine in Baltimore, Nedelcovych’s group developed an OaR drug protector that is specific for PSMA called JHU-2545 [26], with a chemical and biological action that is similar to that of the drugs used in our study. A comparison of single-patient pre-therapy using ^68^Ga-PSMA-11 with and without JHU-2545 showed a SUV_max_ reduction of 41.8% in parotid glands and 31.4% in kidneys. In 2 patients, a 15-min pre-therapy drug administration revealed a reduced uptake of 26% for parotid glands (0.38 and 0.37 mGy/MBq vs. 1.44 mGy/MBq [range = 0.72–1.90] control group) and 56% for kidneys (0.43 and 0.45 mGy/MBq vs. 0.78 mGy/MBq [0.50–0.99]). Although the number of patients was too small to evaluate the drug as an organ protector, Nedelcovych’s results are consistent with our findings on parotid gland sparing and clearly demonstrate that highly specific PSMA organ protectors could be highly advantageous. As they are different from JHU-2545 that was developed in a laboratory and has yet to be validated, the drugs used in our study are commercially available, (relatively) inexpensive and ready for clinical use.

Our study has a number of limitations.

The patient cohort would need to be increased to provide more robust data and further confirm the role of organ protectors of the administered drugs.

The generally poor performance status of patients enrolled in the treatment protocol also affected the number who were able to participate in the dosimetric protocol.

However, this is also true for the other published studies, most of which carried out a dosimetric analysis on a patient cohort that is comparable with ours.

Another factor affecting our analysis was organ overlap, such as high intestinal uptake or lesion overlap, which may compromise the obtained results. Although laxatives were administered to the majority of our patients before and shortly after treatment infusion (7/9), transient high intestinal uptake was still observed in post-infusion images. Laxative administration schemes (i.e., extension of drug administration 2–3 h after infusion) could be investigated to further reduce intestine uptake. 

The implementation of fully 3D dosimetry or hybrid techniques (i.e., combination of whole planar dosimetry and one 3D image for space distribution uptake evaluation) could improve the accuracy of absorbed dose evaluation for different organs, especially kidneys and target structures. After this, kidney absorbed dose evaluation could be more accurate using a hybrid approach. 

Despite the above limitations, our results are nevertheless encouraging. With regard to parotid glands, we only administered 2 tablets per treatment cycle. The optimum number of tablets and timing of administration requires a little ‘fine-tuning’ to improve efficacy. Given that the maximum uptake value was observed around 16 h post infusion, the further administration of candies before the maximum uptake time could reduce overall uptake in theory.

## 4. Materials and Methods

### 4.1. Patient Enrolment

Patients with histologically or cytologically confirmed advanced mCRPC (PCWG3 criteria [27]) who were previously treated with docetaxel and abiraterone or enzalutamide were enrolled in the study. Patients were only admitted to the therapeutic phase if the diagnostic PET/CT ^68^Ga-HBED-CC-PSMA-11 images showed significant uptake (tumor to background ratio >2.5) at the metastatic tumor site (or in the primary, when present). The additional inclusion criteria were age ≥ 18 years; Eastern Cooperative Oncology Group (ECOG) performance status <2 [28]; adequate hematological, liver and renal function (absolute hemoglobin ≥ 9 g/dL; neutrophil count (ANC) ≥ 1.5 × 10^9^/L; platelets ≥ 100 × 10^9^/L; bilirubin ≤1.5 x upper normal limit (UNL), alanine aminotransferase (ALT) and aspartate transaminase (AST) < 2.5 × UNL (<5 × UNL in the presence of liver metastases; and creatinine <2 mg/dL). The exclusion criteria were: assessed bone marrow invasion >50%; previous chemotherapy, ^223^Ra radiotherapy treatment ≤ 4 weeks of enrolment; palliative radiotherapy ≤ 2 weeks of enrolment; and persistence of acute toxicities from any prior therapy (grade >1, CTCAE, version 4.03). The study protocol [29] was approved by our Institutional Ethics Committee and written informed consent was obtained from all patients (EudraCT 2016-002732-32, Ethical approval no. 1704 of 26.10.2016, Protocol IRST 185.03).

### 4.2. Radiopharmaceutical Production

National good preparation standards (NBP MN [30]) for pharmaceutical products were followed for ^177^Lu-PSMA-617 production, as required by the European Association of Nuclear Medicine (EANM). DOTA-PSMA-617 was purchased from Endocyte Inc. (3000 Kent Ave, West Lafayette, IN, USA) and 177Lu from PerkinElmer (68 Elm St, Hopkinton, MA, USA) and AAA (LuMark^®^, Weverstraat 17, 5111 PV Baarle-Nassau, The Netherlands). The labeling procedure and quality control of ^177^Lu-DOTA-PSMA-617 compound was performed in the Radiochemistry Laboratory of our institute (Appendix A).

### 4.3. Treatment Procedure

The study design included 2 patient cohorts. Patients who refused or were unfit to undergo treatment with docetaxel received 5.5 GBq per cycle of ^177^Lu-PSMA-617, while patients previously treated with docetaxel (at least 3 cycles) received lower radiopharmaceutical levels ranging from 3.7 GBq to 4.2 GBq per cycle and 3.7–4.2 GBq of ^177^Lu-PSMA-617 were also administered to patients > 75 years old, regardless of previous docetaxel administration. Patients underwent 4 cycles, which were repeated at intervals of 8–12 weeks. Up to 2 additional cycles were administered if there was no toxicity or evidence of disease progression and if, in the opinion of the investigator, further treatment could clinically benefit the patient. The radiopharmaceutical was slowly infused intravenously over 15–30 min in a dedicated room using a dedicated pump system (patent US 7,842,023 B2).

### 4.4. Renal and Salivary Gland Protection

To reduce salivary gland uptake, 2 folic polyglutamate tablets were orally administered to patients combined with an ice pack placed at each side of the neck 30 min before and during infusion. To preserve kidney functionality, a 10% mannitol solution in 500 mL was infused before and after ^177^Lu-PSMa-617 injection, 250 mL 30 min before therapy and 250 mL one hour after therapy [18,31].

### 4.5. Image Acquisition and Analysis

The gamma emission of ^177^Lu (113 and 208 KeV, relative abundance of 6% and 11%, respectively) enabled us to monitor the radiopharmaceutical biodistribution during the therapeutic phase. Dosimetry evaluation was performed during the first or second treatment cycle. 

Planar whole body images (WBI) were acquired at 30–60 min, 16–24 h, 36–48 h and 120 h post infusion (Figure 1). Imaging was performed on a Discovery NM/CT 670 scanner (International General Electric, General Electric Medical System, Haifa, Israel). The dual-head gamma camera was equipped with 3/8”-thick NaI(Tl) crystals. Anterior and posterior views were acquired with 7 cm/min scan speed, an energy window of 20% applied around the dominant photon peak at 208 keV and a medium-energy high resolution (MEHR) collimator. Two additional energy scatter windows at 175 keV (10% width) and 238 keV (10% width) were used to apply the triple energy window-scatter correction to both posterior and anterior images. 

The first WBI was performed before bladder voiding because the total counts in this image were intended as a surrogate of the effective injected activity and were used to calculate the time–activity curves. The WBI was a 256 × 1024 pixel matrix with pixel dimensions of 2.21 x 2.21 cm. Body contouring to maintain a fixed detector-to-patient distance during image acquisition between scans was not used. 

For attenuation correction, a pre-infusion WBI transmission scan was performed in anterior projection with a sealed flood source (^57^Co) providing transmission and blank images, using low-energy high resolution (LEHR) collimators. ROIs for different organs (i.e., kidneys, abdomen, parotid glands, liver) were identified on both transmission and blank images. Furthermore, the water equivalent thickness was evaluated as:

(1) $$z=\mu_{\left(57\mathrm{Co}\right)}\times \;\ln\left(\frac{I_{\mathrm{transnission}}}{I_{\mathrm{blank}}}\right)$$

where $I_{\mathrm{transnission}}$ and $I_{\mathrm{blank}}$ were average counts on transmission and blank images, respectively; and $\mu_{\left(57\mathrm{Co}\right)}$ the attenuation coefficient for ^57^Co emissions. 

For activity quantification, ROIs were contoured on the first image for the whole body, kidneys, parotid glands and liver. Background regions for each ROI on both anterior and posterior images were also drawn close to the same body region, avoiding the overlap with other structures experiencing uptake (i.e., bladder, intestine). Sequential images were registered in the cranio-caudal direction and ROIs were propagated to all images. If needed, manual adjustments were performed to reduce organ mismatch among sequential images. In the event of an overlap between kidney and high intestinal uptake, the kidney contour was corrected on the single image to eliminate the intestinal uptake [32].

The source organ activity at a particular time-point was estimated by applying the conjugate projection method [33] according to the following equation:

(2) $$A_{\mathrm{ROI}}=\sqrt{\frac{I_{A}{*I}_{P}}{e^{-\mu_{\left(177\mathrm{Lu}\right)}\times z}}}\times e^{-\tau \times \Delta t}$$

where I_A_ and I_p_ were the mean counts per seconds [cps] in the ROI in anterior and posterior views, respectively; $\mu_{\left(177\mathrm{Lu}\right)}$ was the attenuation correction factor for ^177^Lu; τ was the mean ^177^Lu half-life; and Δt time was the difference between infusion and WBI acquisition. For paired organs (kidneys and parotid glands), the mean value was calculated between the left and right organs and a single time–activity curve was obtained.

After this, biological time–activity curves %IA(t) were calculated normalizing A_ROI_ values at each time-point to the total cps in the whole body ROI drawn in the first WBI image ($A_{\mathrm{WBI}}$), which was considered as a reference for the total effective injected activity.

### 4.6. Blood Sample Acquisition and Analysis

Blood samples (2-cc volume) were collected before each WBI acquisition. The samples were analyzed with High-Purity-Germanium (HPGe, ORTEC, Ametek, TN, USA) Radiation Detector (24 h acquisition). The measured activity was corrected for decay and biological time–activity curves were calculated for blood samples. 

### 4.7. Dosimetric Analysis

The dose evaluation was performed according to the MIRD formalism [33,34,35] with OLINDA/EXM software (v 1.1, 2201 West End Ave, Nashville, TN, USA) [36]). Biological time–activity curves were fitted with mono- or bi-exponential curves, depending on the observed kinetic characteristics. Adult male OLINDA/EXM phantom organ models were used for kidneys, liver and whole body. Sphere model was used for parotid glands, assuming unit density composition (i.e., water) [37]. A WBI CT scan was used to evaluate the single organ weight for each patient and for phantom organ scaling (contouring performed on MimVista (v 6.6.5, MIM software, 25800 Science Park Drive-Suite 180, Cleveland, OH, USA).

For red marrow dosimetry, a fast equilibrium in terms of uptake between blood and RM extracellular fluid was assumed [38]. A bi-exponential curve model was used for wash-out fitting. The total blood volume [cc] was evaluated based on single-patient height h [cm] and weight w [g] [39]

(3) $$\mathrm{Bw}=\left(0.3669\times h^{3}\right)+\left(0.03219\times w\right)+0.6041$$

After this, blood mass was calculated with a mean blood density of 1.06 g/cc [39]. Finally, red marrow mass was evaluated with a 0.224 blood/red marrow mass ratio for the standard adult male [36]. The red marrow model of OLINDA/EXM software was used for absorbed dose calculation. The remainder of the body was also considered.

### 4.8. Statistical Analysis

Data were compared to values reported in the literature. The studies by Baum et al. (2015) [7], Delker et al. (2016) [8] and Kabasakal et al. (2015) [9] were considered for the kidneys, parotid glands and whole body dosimetry comparison. Data comparison was performed in terms of both median difference (Mann-Whitney-Wilcoxon) and data distribution (Kolmogorov-Smirnov). R-software (v 3.5.2, https://www.r-project.org/) was used for statistical analyses and box plots were used for graphical data comparison.

## 5. Conclusions

Our results show that the treatment protocol is safe and that the organ protector used could help to reduce any out-of-target uptake. Further optimization of drug quantity and scheme administration is needed to enhance organ preservation. The proposed drug protectors are safe, commercially available, inexpensive, well tolerated, non-invasive and easy to administer in clinical practice. Our data represent a promising starting point for reducing the side-effects in view of more effective therapies, such as alpha-emitter-based radioligands.

## Figures and Tables

**Table 1:** Patient and main treatment characteristics. NA = not available.

Patient	Age [y]	Gleason Score [19]	Injected Activity [GBq]	Bone Marrow Dosimetry	Main Lesion Sites
1	64	NA	5.5	No	Bone
2	85	NA	4.4	No	Bone/tissue
3	71	8	4.4	Yes	Bone/tissue
4	66	9	4.4	Yes	Bone/tissue
5	68	7	5.5	Yes	Bone/tissue
6	53	10	4.4	Yes	Bone
7	62	9	5.5	Yes	Bone/tissue
8	76	8	5.5	No	Bone
9	70	8	5.5	Yes	Bone

**Table 2:** Effective half-life [h] of considered organs. SD = standard deviation.

Patient	Parotid Glands [h]	Kidneys [h]	Liver [h]	Red Marrow [h]	Whole Body [h]
1	35.4	50.7	62.9	-	78.0
2	41.5	12.2	18.1	-	31.9
3	34.6	28.8	30.0	8.7	66.2
4	25.6	21.8	12.5	7.7	31.6
5	30.3	31.4	16.2	3.1	40.1
6	60.7	57.9	59.9	2.5	77.4
7	28.1	39.8	21.6	14.7	33.6
8	29.7	29.4	25.4	-	33.5
9	33.0	80.6	54.9	11.4	79.7
Median (range)	33.0 (25.6–60.7)	31.4 (12.2–80.6)	25.4 (12.5–62.9)	8.2 (2.5–14.7)	40.1 (31.6–79.7)
Mean (SD)	35.4 (10.6)	39.2 (20.9)	33.5 (20.0)	8.0 (4.7)	52.4 (22.2)

**Table 3:** Results of dosimetric study in terms of mGy/MBq (normalized to injected activity). Whole body and kidney model was used in OLINDA/EXM software, while sphere model of unit density was used for parotid gland modeling. SD = standard deviation.

Patient	Parotid Glands [mGy/MBq]	Kidneys [mGy/MBq]	Liver [mGy/MBq]	Red Marrow [mGy/MBq]	Whole Body [mGy/MBq]
1	1.23	0.70	0.11	-	0.113
2	2.63	0.38	0.10	0.044	0.035
3	0.79	1.07	0.15	-	0.044
4	0.41	0.26	0.05	0.023	0.018
5	0.48	0.77	0.14	0.061	0.038
6	0.65	0.50	0.05	0.067	0.088
7	0.37	1.00	0.13	0.036	0.027
8	0.41	0.78	0.19	-	0.033
9	0.33	0.63	0.53	0.033	0.043
Median (range)	0.48 (0.33–2.63)	0.70 (0.26–1.07)	0.13 (0.05–0.53)	0.044 (0.023–0.067)	0.038 (0.018–0.113)
Mean (SD)	0.81 (0.74)	0.67 (0.27)	0.16 (0.15)	0.044 (0.017)	0.049 (0.031)

## Appendix A

The manual radiosynthesis of ^177^Lu-DOTA-PSMA-617 can be divided into five steps:

- **Dose Calibrator Response Verification**: Prior to each production, the background and instrument response are measured using a certified source of ^137^Cs (NuklearMedizin, Dresden, Germany) with known activity; the percentage of deviation between the measured value and the expected value is measured and recorded. The deviation must never be greater than 5%.
- **Measure of the incoming ^177^Lu activity**: the arrival vial of ^177^LuCl_3_ is measured in the dose calibrator, after which a mixture is prepared containing a sufficient quantity of DOTA-PSMA-617 (calculated with a ratio of 0.9 μg/mCi) and a sufficient volume of the buffer solution to maintain the reaction pH at 5.0.
- **Complexation Reaction ^177^Lu-DOTA-PSMA-617**: the reaction mixture is heated to 100 degrees for 8 min.
- **Transfer and dilution**: the radiopharmaceutical is then transferred into a 30 mL bottle (Drytec, GE Healthcare Buchler, 38110, Braunschweig, Germany), through a sterile line equipped with 0.22-μm ventilated sterilizing filter (Millex-GV Syringe Filter Unit, 25 mm PVDF (low protein binding membrane)). This solution is then diluted with physiological solution until a final volume of 17-22 mL is obtained.
- **Measurement of the final activity and concentration calculation**: the final vial is measured in a dose calibrator with the appropriate geometry to evaluate the yield and the final activity available for the patient-specific doses.
